# Gait kinematics of the hip, pelvis, and trunk associated with external hip adduction moment in patients with secondary hip osteoarthritis: toward determination of the key point in gait modification

**DOI:** 10.1186/s12891-019-3022-1

**Published:** 2020-01-06

**Authors:** Hiroshige Tateuchi, Haruhiko Akiyama, Koji Goto, Kazutaka So, Yutaka Kuroda, Noriaki Ichihashi

**Affiliations:** 1 Department of Preventive Physical Therapy, Human Health Sciences, Graduate School of Medicine, Kyoto University, 53 Kawara-cho, Shogoin, Sakyo-ku, Kyoto, 606-8507 Japan; 2 Department of Physical Therapy, Human Health Sciences, Graduate School of Medicine, Kyoto University, Kyoto, Japan; 3 Department of Orthopaedic Surgery, School of Medicine, Gifu University, Gifu, Japan; 4 Department of Orthopaedic Surgery, Graduate School of Medicine, Kyoto University, Kyoto, Japan; 5 Department of Orthopaedic Surgery, Osaka Red Cross Hospital, Osaka, Japan

**Keywords:** Hip osteoarthritis, Gait, Joint moment, Pelvis, Trunk

## Abstract

**Background:**

A larger daily cumulative hip loading, which is the product of the external hip adduction moment (HAM) impulse during gait and the number of steps per day has been identified as a factor associated with the progression of secondary hip osteoarthritis (OA). The cause of the increased HAM impulse in patients with hip OA has not been identified. The purpose of this study was to identify the gait parameters associated with HAM impulse during gait in patients with secondary hip OA.

**Methods:**

Fifty-five patients (age 22–65 years) with mild-to-moderate secondary hip OA participated in this cross-sectional study. The HAM impulse during gait was measured using a three-dimensional gait analysis system. To identify the gait parameters associated with HAM impulse, hierarchical multiple regression analysis was performed. The first model (basic model) included body weight and stance phase duration. The second models included gait parameters (gait speed; ground reaction force [GRF] in frontal plane; and hip, pelvic, and trunk angle in frontal plane) and hip pain in addition to the basic model.

**Results:**

Body weight and stance phase duration explained 61% of the variance in HAM impulse. In the second model, which took into account body weight and stance phase duration, hip adduction angle (9.4%), pelvic tilt (6.5%), and trunk lean (3.2%) in addition to GRF explained the variance in the HAM impulse. Whereas larger hip adduction angle and pelvic tilt toward the swing limb were associated with a larger HAM impulse, larger trunk lean toward the stance limb was associated with smaller HAM impulse.

**Conclusion:**

In patients with excessive hip adduction and pelvic tilt toward the swing limb during gait, gait modification may contribute to the reduction of hip joint loading.

## Background

From the standpoint of biomechanics, overloading of the joint has been considered one of the main causes of the onset and progression of osteoarthritis (OA) [1]. For hip OA, larger daily cumulative hip loading, which is the product of the external hip moment impulse during gait particularly in the frontal plane (i.e., external hip adduction moment [HAM] impulse) and the mean number of steps per day, is a risk factor for subsequent radiographic progression of the secondary hip OA [2]. Therefore, although maintaining daily physical activity is recommended for patients with OA [3], it may be important to control physical activity to prevent excessive daily cumulative hip loading and hip OA progression.

Reduction of both the HAM impulse during gait and the number of steps is required for reducing the daily cumulative hip loading. The number of steps can be evaluated using a pedometer, and a patient may self-regulate under the advice of a clinician. However, for both patients and clinicians, it is difficult to control the HAM impulse which is considerably larger than the hip moment impulse in the sagittal and horizontal planes. To reduce the HAM impulse, it is imperative to first identify the factors that affect it. Identifying these factors in patients before they reach the terminal-stage of hip OA would be an important step toward preventing hip OA progression.

As the external HAM is primarily the product of the frontal plane component of the ground reaction force (GRF) magnitude and its lever arm between the hip joint center of rotation and the GRF vector [4], both GRF magnitude and gait kinematics may affect the HAM. Body weight has a significant effect on the variance in HAM during gait [5]. As the moment impulse is temporally integrated with the moment curve (area under the curve) [2], the duration of the stance phase affects the HAM impulse. In knee OA, body weight, GRF magnitude, trunk lean during gait, and knee adduction angle at the same instance in time as the peak moment have been reported to be associated with the peak external knee adduction moment [6–8]. Increased knee adduction angle and trunk lean can alter the knee adduction moment by changing the lever arm length via displacement of the knee joint center of rotation laterally and changing the direction of the GRF vector. HAM generally has bimodal peaks even in patients with hip OA and the first peak is often greater than the second [2, 9]. In patients with hip OA, the peak vertical ground reaction force shows a linear relationship with the first peak of HAM [9]. However, gait kinematics related to the HAM impulse have not been identified in patients with hip OA. Therefore, we have not been able to determine a suitable target in gait modification training to reduce hip joint loading. Various modifications of gait patterns related to HAM impulse shown in patients with symptoms and impairments of the hip joint may be different from the theoretical speculation. The gait parameters associated with HAM impulse in actual patients with hip OA must be verified.

The primary aim of this study was to identify the gait parameters associated with HAM impulse during gait with adjustment for body weight and stance phase duration in patients with mild-to-moderate secondary hip OA. The secondary aim was to clarify the relationship among ground reaction force and gait kinematics during gait to gain insight into the mechanism underlying gait changes as basic information for gait modification in patients with hip OA. Given that changes in the alignment of the hip, pelvis, and trunk can change the inclination of the GRF vector and the distance between the GRF vector and the center of hip joint [4], we hypothesized that larger hip adduction and pelvic and trunk tilts toward the swing limb would be associated with a larger HAM impulse by increasing the lever arm between the hip joint center of rotation and the GRF vector.

## Methods

### Participants

Patients were selected from among non-surgical outpatients in the Department of Orthopaedic Surgery at Kyoto University Hospital. Patients aged 20 years and older, with secondary hip OA were recruited continuously from April 2013 to June 2015. Secondary hip OA is defined as OA resulting from a traumatic injury, specific anatomical deformities in the joint, or specific abnormalities of the cartilage extracellular matrix [10]. A total of 55 patients were included in our study. The inclusion criteria were as follows: (1) a diagnosis of pre-hip OA (acetabular dysplasia with no other abnormal radiographic findings), early-hip OA (slight joint space narrowing [2 mm or more] and abnormal subchondral sclerosis), or advanced-stage hip OA (marked joint space narrowing [less than 2 mm] with or without cysts or sclerosis) [11] and (2) ability to walk without any assistive device in daily life. The exclusion criteria were as follows: (1) patients with terminal-stage hip OA (gross loss of joint space with a width of 15 mm or more) [11]; (2) a history of previous hip surgeries (e.g., osteotomy and arthroplasty); and (3) neurologic, vascular, or other conditions that affected gait or activities of daily living. The proportion of patients in each stage of hip OA were as follows: pre- (*n* = 16, 29.1%), early- (*n* = 25, 45.5%), and advanced-stage (*n* = 14, 25.5%). The median value of hip pain (visual analogue scale) and the Harris hip score of the patients included in this study was 43 mm and 91 points, respectively (Table 1). Our sample was biased in gender (6.9% were males); therefore, only female patients were included in this study to reduce the heterogeneity of the study population. The side with more severe radiographic OA change was used for the analysis. In this study, we used the same cohort as that in a previous study [12]. All participants provided written informed consent, and the protocol was approved by the Ethics Committee of the Kyoto University Graduate School and Faculty of Medicine (protocol identification number: E1683).

**Table 1:** Participants’ demographic and clinical data (*n* = 55)

	Mean	SD	Minimum	Maximum
Age, years	47.9	10.4	22	65
Weight, kg	55.2	9.9	38.5	79.9
Height, cm	156.9	5.6	145.4	172.1
Minimum JSW, mm	3.5	1.3	0.4	6.0
	Median	Interquartile range	Minimum	Maximum
Hip pain (VAS)^a^, mm	43	16–68	0	97
Harris hip score (total 100 points)^a^	91	80–96	64	100

(Footnotes for Table 1) *JSW* Joint space width, *VAS* Visual analogue scale. ^a^ Data was non-normally distributed

### Gait analysis

Gait analysis was conducted using an 8-camera Vicon motion system (Vicon Motion Systems Ltd., Oxford, England) at a sampling rate of 200 Hz with a fourth-order Butterworth low-pass filter with a 6 Hz cutoff and force plates (Kistler Japan Co., Ltd. Tokyo, Japan) at a sampling rate of 1000 Hz with a low-pass filter (20 Hz). A total of 26 reflective markers were placed by a single experienced examiner to minimize the marker placement error. Each segment was composed of marker sets as follows: Trunk, the 7th cervical spinous process, the 10th thoracic spinous process, the jugular notch, the xiphoid process, and the bilateral acromioclavicular joints; Pelvis, bilateral anterior and posterior superior iliac spine; Thigh, the superior aspect of the greater trochanter and the medial and lateral femoral condyles; Shank, the medial and lateral femoral condyles and the medial and lateral malleoli; Foot, the heel, the head of the 1st and 5th metatarsal, and the medial and lateral malleoli. The three-dimensional (3D) angles of the hip, pelvis, and trunk, as well as 3D external joint moments of the hip were calculated using BodyBuilder software (Vicon Motion Systems Ltd., Oxford, England). The angles of the pelvis and trunk were calculated in a global coordinate system. We used the peak values of the joint/segment angle as parameters so that they could be used as the key points in gait modification.

The participants were clothed in close-fitting shorts and T-shirts and asked to walk barefoot along a 7-m walkway with 4 force plates embedded in the center at their usual speed after several practice trials. We used the following variables on the affected side as gait-related factors: gait speed; stance phase duration; magnitude of the GRF in the frontal plane (scalar); peak angle of the hip, pelvis, and trunk in the frontal plane; and external HAM impulse during a stance phase. The mean values from three successful trials were calculated and used for analysis.

### Radiographical and clinical measurements

The stage of the hip OA was evaluated using minimum joint space width (JSW) in a digital supine anteroposterior radiograph of the pelvis in all patients. The radiograph was obtained in a standardized manner by the same skilled radiology technicians within 1 month before the gait analysis. Measurement of the minimum JSW was performed by a single experienced examiner. Images were reviewed and measured using Centricity Enterprise Web, version 3.0 (GE Health care, Buckinghamshire, England). The JSW was measured in 0.1 mm increments at three locations, namely, the lateral margin of the subchondral sclerotic line, apical transection of the weight-bearing surface by a vertical line through the center of the femoral head, and the medial margin of the weight-bearing surface bordering on the fovea. If the minimum JSW was found in regions aside from the three locations in the weight-bearing area, the JSW of the narrowest point was also recorded as a fourth measurement. Minimum JSW was defined as the smallest of the three or four measurements [13]. The intra-rater reliability (intra-class correlation 1,1) of our JSW measurement for 20 randomly selected radiographs was 0.99.

Additionally, the intensity of hip pain during daily life and functional status of the patients were assessed using a 100-mm visual analogue scale and Harris hip score [14], respectively, on the same day as gait analysis.

### Statistical analysis

To identify the gait parameters associated with HAM impulse, we performed hierarchical multiple regression analysis. We chose this analysis to determine the unique contribution of each gait parameters to the total variance explained by the model. The first model (basic model) included only body weight and stance phase duration to verify the influence of each gait parameters after including factors that clearly influence the HAM impulse. The second models included the gait parameters (gait speed; GRF in the frontal plane; and peak angles of the hip, pelvic, and trunk in the frontal plane) in addition to the basic model. Additionally, we also examined a model including hip joint pain because it has been reported that a change in joint pain because of treatment is directly related to the change in joint moment during gait [15, 16]. Multicollinearity was examined using the absolute values of the correlation coefficients (|r|), where |r| > 0.7 indicated multicollinearity [17] and the variance inflation factor with a threshold of 5. A *P* value < 0.05 was considered statistically significant. SPSS version 24.0 (IBM Japan Ltd., Tokyo, Japan) was used for statistical analyses.

## Results

**Fig. 1:**
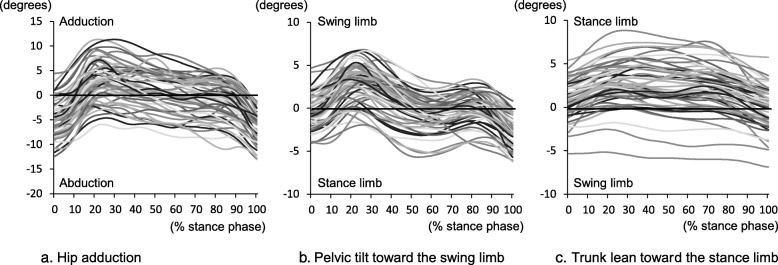
Waveforms of hip adduction-abduction angle, pelvic tilt, and trunk lean angles in the frontal plane during the stance phase for all patients. Positive values represent hip adduction (**a**), pelvic tilt toward the swing limb (**b**), and trunk lean toward the stance limb (**c**)

Gait parameters of the 55 patients are presented in Table 2. Individual kinematic patterns of the hip, pelvis, and trunk during a stance phase are shown in Fig. 1.

**Table 2:** Participants’ gait parameters including hip, pelvis, and trunk kinematic and kinetic variables in the frontal plane (*n* = 55)

	Mean	SD	Minimum	Maximum
Gait speed, meters/seconds	1.14	0.16	0.75	1.53
Ground reaction force (Frontal plane), N	612.2	107.9	400.5	910.4
Stance phase duration, seconds	0.51	0.04	0.41	0.63
Peak hip abduction angle, degrees	7.2	2.9	1.4	13.4
Peak hip adduction angle, degrees	3.6	3.8	−5.3	11.4
Peak pelvic tilt angle (toward stance limb), degrees	3.1	1.6	0.1	7.1
Peak pelvic tilt angle (toward swing limb), degrees	3.2	2.2	−1.9	7.3
Peak trunk lean angle (toward stance limb), degrees	3.1	2.6	−5.1	8.9
Peak trunk lean angle (toward swing limb), degrees	0.2	2.3	−5.4	6.9
Hip adduction moment impulse, Nm•seconds	22.7	7.3	9.1	44.1

Results of the multivariable linear regression analysis are presented in Table 3. The basic model,which included body weight and stance phase duration, was able to explain 61% of the variance in HAM impulse (*P* <  0.001). A larger body weight and longer stance phase duration were independently associated with a larger HAM impulse.

**Table 3:** Regression models for hip adduction moment impulse as the dependent variable (n = 55)

Independent variables	Unstandardized coefficients B	Standardized coefficients β	*P*–value for variable	95% confidence interval for B	R	R^2^	Adjusted R^2^	Adjusted R^2^ change
Basic model					0.787	0.620	0.606	0.606
Body weight, kg	0.473	0.644	**< 0.001**	0.340, 0.606				
Stance phase duration, seconds	46.959	0.288	**0.002**	17.359, 76.560				
Separate models with gait parameters (weight + stance phase duration + following gait characteristics)								
Gait speed, meters/seconds	7.264	0.158	0.169	−3.197, 17.724	0.796	0.634	0.613	0.007
Ground reaction force (Frontal plane)^a^, N	0.040	0.599	**< 0.001**	0.028, 0.052	0.773	0.598	0.583	0.347
Peak hip abduction, degrees	−0.311	− 0.125	0.149	−0.737, 0.115	0.797	0.635	0.614	0.009
Peak hip adduction, degrees	0.612	0.325	**< 0.001**	0.317, 0.907	0.846	0.719	0.700	0.094
Peak pelvic tilt (toward stance limb), degrees	−0.118	−0.027	0.775	−0.945, 0.708	0.788	0.621	0.598	−0.007
Peak pelvic tilt (toward swing limb), degrees	0.878	0.266	**0.001**	0.354, 1.401	0.830	0.689	0.671	0.065
Peak trunk lean angle (toward stance limb), degrees	−0.558	−0.198	**0.022**	−1.033, −0.083	0.811	0.658	0.637	0.032
Peak trunk lean angle (toward swing limb), degrees	0.265	0.084	0.330	−0.276, 0.806	0.792	0.627	0.605	0.000
Separate model with hip pain (weight + stance phase duration + hip pain)								
Hip pain (VAS), mm	−0.022	−0.083	0.375	−0.071, 0.027	0.791	0.626	0.604	0.006

(Footnotes for Table 2) ^a^ Body weight was excluded from independent variables

All of the regression models are *P* < 0.001. Bolded values are statistically significant

**Fig. 2:**
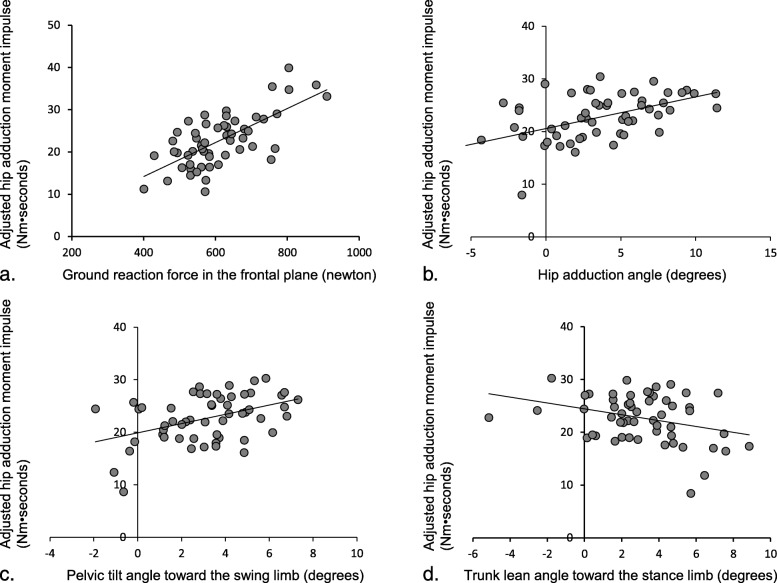
Adjusted scatter plots between hip adduction moment impulse and ground reaction force in the frontal plane (**a**), hip adduction angle (**b**), pelvic tilt angle toward the swing limb (**c**), and trunk lean angle toward the stance limb (**d**). Value of the vertical axis represents hip adduction moment impulse adjusted for stance phase duration (**a**), and hip adduction moment impulse adjusted for body weight and stance phase duration (**b**, **c**, and **d**)

In the second models, larger hip adduction angle and pelvic tilt toward the swing limb were statistically significantly associated with larger HAM impulse, explaining 9.4 and 6.5% of the variance in the HAM impulse, respectively (Table 3). According to the unstandardized coefficient B, a 1 degree increase in hip adduction angle or pelvic tilt toward the swing side can increase the HAM impulse by 0.612 or 0.878 Nm•seconds, respectively. The second model including body weight and stance phase duration with hip adduction angle or pelvic tilt toward the swing limb explained 70 and 67% of the variance in HAM impulse, respectively. Since multicollinearity was indicated between GRF in the frontal plane and body weight (*r* = 0.916, *P* <  0.001), regression analysis was performed for GRF in the frontal plane adjusted only for stance phase duration. Larger GRF in the frontal plane was statistically significantly associated with larger HAM impulse (Fig. 2). Peak trunk lean toward the stance limb was negatively statistically significantly associated with HAM impulse, explaining 3.2% of the variance in the HAM impulse. Larger trunk lean toward the stance limb was associated with smaller HAM impulse (Fig. 2). Hip pain was not significantly associated with HAM impulse.

## Discussion

The results of our study provide support for our hypothesis, as we demonstrated that hip adduction and pelvic tilt toward the swing limb are important kinematic factors for HAM impulse during gait. The body weight and stance phase duration may explain 61% of the variance in HAM impulse. Nevertheless, this study showed significant contributions by hip and pelvic kinematics during gait to the HAM impulse. Although these findings are theoretically understandable [18] and the relationship between peak HAM and kinematics of the pelvis and trunk have been reported in patients with gluteal tendinopathy [19], it is clinically significant that this study has, for the first time, explained the variance in HAM impulse by noting the variation in the gait in actual patients with hip OA. We have chosen to include patients with acetabular dysplasia and hip OA because in a context where the focus of treatment for OA has shifted from palliation to prevention [20], it is reasonable to include patients with acetabular dysplasia as it is regarded as a pre-osteoarthritis condition in the course of hip OA progression.

Hip adduction angle had the greatest influence on HAM impulse among gait kinematics, explaining 9.4% of the variance in HAM impulse. The association between hip adduction angle during gait and HAM impulse (adjusted R^2^: 0.700) is comparable to, or slightly stronger than, the bivariable association between the knee adduction angle and peak external knee adduction moment (KAM) during gait in healthy individuals (adjusted R^2^: 0.566) [6], asymptomatic individuals with normally or varus-aligned knees (adjusted R^2^: 0.467) [21], and patients with knee OA (adjusted R^2^: 0.489) [22]. As a motion pattern, most of the patients’ hip joints showed a pattern of movement in the adduction direction during the loading response after initial contact, in the same manner as that in healthy individuals [23]. However, there were many inter-individual variations in the hip joint position of hip abduction/adduction during the stance phase (Fig. 1). Relatively medially shifted contact of the lower limb with the floor during hip adduction can displace the origin of the GRF medially, relative to the center of the hip joint. Thus, larger hip adduction would increase the HAM impulse by increasing the lever arm between the hip joint center and GRF vector. In muscle-driven gait simulations, an increase in hip adduction was shown to be the most influential kinematic affecting the increase in peak HAM and the hip contact force [18]. In gait analysis in the context of hip joint overloading, excessive hip adduction should be given substantial attention.

We observed that pelvic tilt toward the swing limb was also a factor related to the larger HAM impulse. The excessive pelvic tilt toward the swing limb during gait is widely known as Trendelenburg gait. The Trendelenburg gait has been confirmed not only in patients with hip OA but also in patients with gluteal tendinopathy [19]. Allison et al. [19] reported that the contralateral pelvic drop at each time point were related to the variation of the first and second peaks of HAM which were greater in patients with gluteal tendinopathy than in the controls. The mean pelvic tilt angle of patients with hip OA was 3.2°, which was not vastly different from that of healthy individuals (approximately 4°, the value of healthy individuals at almost the same gait speed as in the patients in our study) [24]. However, some patients showed excessive pelvic tilt toward the swing limb with a maximum angle of 7.3°. The pelvic tilt toward the swing limb can displace the mass of the upper body to the swing side even if it is not accompanied by a trunk lean. Consequently, the center of mass of the body is displaced to the swing side, and the GRF vector is also displaced away from the hip joint. The larger HAM impulse would attribute to the change in the direction of the GRF vector associated with pelvic tilt. Given that the excessive pelvic tilt toward the swing limb is commonly observed in patients with hip disease, pelvic tilt toward the swing limb is a key point in clinical gait observation and modification in the sense that it can lead to an increase in the HAM impulse.

On the other hand, trunk lean toward the stance limb was associated with smaller HAM impulse, although its contribution was as small as 3.2%. We hypothesized that trunk lean toward the swing limb is associated with larger HAM impulse. Nevertheless, there were only a few patients in this study who showed trunk lean toward the swing limb (Fig. 1). Trunk lean toward the stance limb moves the GRF vector closer to the hip joint by displacing the center of mass of the body to the stance limb. Consequently, HAM impulse can be reduced. In patients with knee OA, trunk lean toward the stance limb has a significant effect on the reduction in KAM [6, 25]. Trunk lean is used as one of the gait modification strategies for reducing KAM [26]. In patients with hip OA, trunk lean toward the affected side tends to increase during gait [9, 27, 28], especially in patients with hip abductor muscle weakness that show significant trunk lean [29]. Trunk lean may be an adaptive strategy to reduce hip loading and pain by decreasing HAM impulse However, although it was observed only in a few cases, HAM impulse was generally large in patients whose trunk leaned toward the swing limb during the stance phase (Fig. 2). For such rare patients, clinicians need to carefully observe trunk lean toward the swing limb as a potential factor affecting HAM impulse.

Interestingly, hip pain was not a factor in explaining the variance in the HAM impulse. The results are inconsistent regarding the relationship between hip pain and kinetic variables in the hip joint during gait. Hurwitz et al. [30] reported that an increased level of hip pain correlated with decreased hip extension moment. Conversely, Zeni et al. [29] have confirmed that there were no gait parameters, including hip moment, associated with hip pain. Moreover, previous prospective cohort studies have shown that the HAM impulse is associated with radiographical (i.e., structural) hip OA progression but not with worsening of hip pain [2, 31]. The relationship between hip pain and gait biomechanics may be modified by various factors such as the condition of joint structures and difference in compensatory strategies for gait using parts other than the hip joint such as the trunk; therefore, hip pain might not be directly related to the HAM impulse. Furthermore, in the present study, the inclusion of younger patients with little hip pain and disability may account for the lack of correlation between hip pain and the HAM impulse.

It is notable that the magnitude of HAM is not necessarily larger in patients with hip OA compared with healthy individuals. Previous studies have shown that HAM is not significantly different between the patients with hip OA and healthy individuals [9, 32], or rather HAM of patients with hip OA is smaller than that of the healthy individuals [30, 33]. However, joint degeneration in patients with secondary hip OA who have morphologic abnormalities such as dysplasia may be adversely affected even if the magnitude of hip loading is equal to or inferior to that of healthy individuals. This is because patients with hip OA generally have a decreased cartilage contact area than do healthy individuals [34], as well as damaged articular cartilage and labrum [35]. Indeed, in the secondary hip OA group, a higher cumulative hip loading related to a large HAM impulse is a risk factor for narrowing of the hip joint space [2]. Thus, identifying the gait parameters related to the increase in HAM impulse is critical to establish gait modification training aimed at preventing hip OA progression.

This study has several limitations. Patients with terminal-stage hip OA were excluded from this study in order to render the findings applicable to the prevention of hip OA progression. However, gait kinematics and kinetics are different between patients with mild-to-moderate hip OA and patients with severe hip OA [36]. Therefore, the results of this study may not be generalizable to patients with terminal-stage hip OA. It is difficult to elucidate causal relationships as this study was a cross-sectional study. Thus, it remains to be firmly established whether HAM impulse during gait in daily life is reduced by improving hip joint adduction and pelvic tilt with gait modification. On the basis of a prospective cohort study showing an association between hip OA progression and gait biomechanics [2], HAM impulse was adopted as a dependent variable. However, other variables such as peak or mean of HAM during the stance phase may also be important factors associated with hip joint loading. Additionally, HAM is an indirect measure of hip joint loading, although HAM is strongly correlated to hip contact force [18]. It is necessary in the future to analyze hip joint force in the model including patient-specific bone morphology, tension of the muscles and ligaments, and muscle recruitment patterns.

## Conclusion

The larger hip adduction and pelvic tilt toward the swing limb in addition to larger body weight, stance phase duration, and GRF were associated with larger HAM impulse. Although larger trunk lean toward the stance limb contributed to the smaller HAM impulse, excessive pelvic tilt toward the swing limb may inhibit the compensatory trunk lean. It is important for clinicians to reduce excessive hip adduction and pelvic tilt towards the swing limb in order to prevent the progression of hip OA.

## Data Availability

The datasets used and/or analyzed during the current study are available from the corresponding author on reasonable request.

## Abbreviations

GRF: Ground reaction force
HAM: Hip adduction moment
JSW: Joint space width
KAM: Knee adduction moment
OA: Osteoarthritis
